# Genetic variants explain ancestry‐related differences in type 2 diabetes risk

**DOI:** 10.1002/ctm2.70076

**Published:** 2024-11-05

**Authors:** Aaron J. Deutsch, Kirk Smith, Miriam S. Udler

**Affiliations:** ^1^ Diabetes Unit Endocrine Division Department of Medicine Massachusetts General Hospital Boston Massachusetts USA; ^2^ Center for Genomic Medicine Massachusetts General Hospital Boston Massachusetts USA; ^3^ Programs in Metabolism and Medical & Population Genetics, Broad Institute of MIT and Harvard Cambridge Massachusetts USA; ^4^ Department of Medicine Harvard Medical School Boston Massachusetts USA

1

Type 2 diabetes (T2D) is a global epidemic, affecting over 400 million people around the world.^1^ T2D causes devasting complications and is a leading risk factor for ischaemic heart disease and stroke, which are among the top causes of global morbidity and mortality.^1^ Classically, T2D occurs in adulthood in the setting of obesity and insulin resistance. Increasingly, however, T2D is understood to arise from a complex interplay of environmental and genetic factors, leading to heterogeneity in patient clinical presentation and disease course.^2^, ^3^ There have been many attempts to define T2D subtypes using a range of analytic methods, but few efforts have shown real‐world clinical utility or given insight into disease pathophysiology.^4^

**FIGURE 1:**
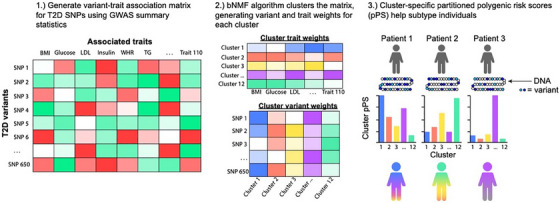
Overview of type 2 diabetes (T2D) genetic clustering pipeline. BMI, body mass index; bNMF, Bayesian non‐negative matrix factorisation; GWAS, genome‐wide association study; LDL, low‐density lipoprotein; pPS, partitioned polygenic score; SNP, single nucleotide polymorphism; TG, triglycerides; WHR, waist‒hip ratio.

Over recent years, advances in large‐scale genome‐wide association studies (GWASs) have uncovered hundreds of genetic variants that modulate T2D risk. This genetic information has the potential to provide insight into disease biology; yet, clinical translation has been limited, often because the strongest genetic associations are not found in protein‐coding regions, which makes it more challenging to identify causal genes and pathways. By leveraging the power of GWAS, our laboratory has developed a complex, high‐throughput approach to define T2D disease mechanisms, which may help to identify T2D patient subtypes^5^, ^6^ (Figure 1). This approach aggregates GWAS results to assess the link between genetic variants and diabetes‐related clinical traits, such as glucose, haemoglobin A1c and body mass index (BMI). We then apply a machine learning method called Bayesian non‐negative matrix factorisation to group together closely related variants and traits into clusters. By analysing the top‐weighted variants and traits in each cluster, we can infer the most likely biological mechanism contributing to that cluster. Notably, this ‘soft’ clustering method allows a given variant or trait to be assigned to more than one cluster.

Most prior genetic analyses have focused on European populations, potentially limiting the applicability for other ancestry groups. To address this limitation, we recently applied our high‐throughput pipeline to investigate T2D clusters using current large, multi‐ancestry genetic studies.^7^ Through this approach, we confirmed our previously identified T2D genetic clusters and found three new clusters, yielding a total of 12 clusters. Three clusters were associated with beta cell dysfunction and insulin deficiency, while seven were associated with insulin resistance. Among the insulin resistance clusters, certain clusters were associated with obesity and above‐average BMI, whereas two other clusters were associated with a ‘lipodystrophy‐like’^8^ abnormal fat distribution and below‐average BMI. Furthermore, we demonstrated significant associations between the clusters and clinical phenotypes. For instance, the lipodystrophy‐like genetic clusters were associated with above‐average risk of fatty liver disease, whereas another cluster defined by reduced cholesterol levels was associated with below‐average risk of coronary artery disease.

**FIGURE 2:**
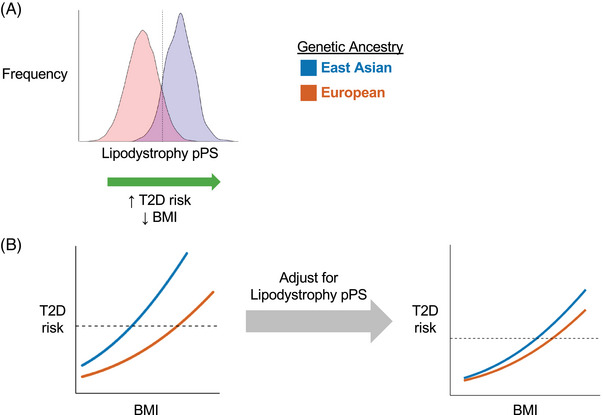
Type 2 diabetes (T2D) genetic clusters modulate relationship between T2D risk and body mass index (BMI). (A) Lipodystrophy‐like genetic risk, as captured by the partitioned polygenic score (pPS), is higher in East Asian compared to European populations. (B) BMI‐adjusted T2D risk is greater in East Asian compared to European populations. However, this difference is diminished after controlling for variation in lipodystrophy‐like genetic risk.

Importantly, we also assessed differences in T2D genetic clusters across ancestry groups. To do this, we aggregated the genetic variants from each cluster to capture each person's cluster‐specific genetic risk. We found that certain clusters were more highly weighted in specific ancestry groups. In particular, the two lipodystrophy‐like genetic clusters contained alleles more frequently seen in individuals with East Asian ancestry, compared to other populations (Figure 2). We hypothesised that these genetic differences might account for varied clinical presentations of T2D.

Notably, it is well established that individuals with East Asian ancestry have a higher risk of T2D at lower BMI levels, compared to those with European ancestry.^9^ This association may be due to an increased risk of a lipodystrophy‐like phenotype, with a greater degree of metabolically unhealthy adipose tissue in East Asian populations.^10^ Indeed, we confirmed that East Asian populations (compared to European populations) had greater susceptibility to T2D at lower BMI levels; however, after controlling for genetic variation in the two lipodystrophy‐like genetic clusters, these ancestry‐specific differences were diminished (Figure 2). Furthermore, we found that these genetic effects were partially mediated through changes in adipose tissue distribution. Individuals with more visceral adipose tissue (compared to subcutaneous adipose tissue) have greater T2D risk; however, population‐specific differences in this relationship were diminished after controlling for variation in the lipodystrophy‐like genetic clusters.

Current guidelines suggest that clinicians may apply different definitions of obesity based on an individual's race and ethnicity (e.g., BMI > 30 kg/m^2^ in White individuals or BMI > 27.5 kg/m^2^ in Asian individuals).^9^ However, using race‐based classification systems is controversial, as it may perpetuate structural racism and may be inaccurate for multiracial individuals. Instead, we propose an individualised approach that focuses on genetic variation. Using this framework, the BMI threshold for obesity is defined based on an individual's risk for T2D, accounting for age, sex and lipodystrophy‐like genetic risk. After adopting this personalised approach, those who had a BMI above their individual obesity threshold had approximately equal T2D risk in both European and East Asian populations.

Overall, these findings demonstrate the ability of genetic information to identify clinically relevant T2D disease mechanisms. By analysing diverse populations, we demonstrated how genetic variation helps to explain phenotypic differences in T2D between European and East Asian ancestry groups. Utilising these genetic variants may allow development of sophisticated decision support tools for clinicians, providing an individualised BMI target to decrease the risk of T2D. Notably, other genetic ancestry groups (e.g., African and South Asian) also have increased risk of T2D at lower BMI thresholds, but variation in our T2D genetic clusters did not account for this observation. This may be due to low representation of non‐European ancestral populations in current GWAS; therefore, it is imperative that researchers continue to broaden the diversity of genetic association studies. Nevertheless, our findings represent an exciting step toward fulfilling the promise of precision medicine in T2D diagnosis and management.
